# Numerical Simulation and Experiment of Electrically-Assisted Incremental Forming of Thin TC4 Titanium Alloy Sheet

**DOI:** 10.3390/ma13061335

**Published:** 2020-03-15

**Authors:** Bo Jiang, Wenbing Yang, Ziyang Zhang, Xifeng Li, Xueping Ren, Yaoqi Wang

**Affiliations:** 1 School of Materials Science and Engineering, University of Science and Technology Beijing, Beijing 10083, China; jiangxiaohang526@126.com (B.J.); rxp33@ustb.edu.cn (X.R.); 2 AVIC Manufacturing Technology Institute, Beijing 10024, China; xiaoqigh@sina.com; 3 Department of Plasticity Technology, School of Materials Science and Engineering, Shanghai Jiao Tong University, Shanghai 200030, China; yangwb@sjtu.edu.cn (W.Y.); zhangziyang@sjtu.edu.cn (Z.Z.)

**Keywords:** electrically-assisted incremental forming, thin TC4 titanium alloy sheet, two-step simulation method, springback

## Abstract

In order to integrally manufacture the large TC4 titanium alloy part, an electrically-assisted incremental forming process is cleverly proposed to solve the traditional hot forming disadvantages of expensive heating furnaces and long cycle period. The two-step simulation method including thermal-electricity coupling simulation and thermo-mechanical coupling simulation was selected to predict the temperature variations and the sheet deformation behaviors. The electrically-assisted incremental forming experiment of thin TC4 titanium alloy sheet was performed. The highest prediction error is 6% for springback angles. The thrice forming at 10.9 A/mm^2^ satisfies the precision requirement of the designed part. Therefore, the two-step simulation method can effectively calculate the electrically-assisted incremental process. The electrically-assisted incremental forming technique is very promising for the integral producing large titanium alloy part.

## 1. Introduction

Titanium alloys are used in a wide range of industries, including aerospace, surgical implants, and fuel cells owing to their excellent properties such as moderately high specific strength, good toughness, and excellent corrosion resistance [1,2,3]. The Ti-6Al-4V (TC4) titanium alloy is most widely used in this alloy family. Due to their poor formability and large springback at room temperature, hot working technique is usually used to produce various kinds of complicated TC4 parts. However, the traditional hot forming process heated in the furnace has the disadvantages of low efficiency, high energy consumption, and complex equipment requirement. The development of advanced electrically-assisted forming (EAF) technology has been a promising method of solving the bad plasticity of titanium alloys at room temperature. It utilizes the electroplastic effects brought by the electric current flowing through metals to assist the deformation of low plasticity materials. Utilizing electric current in the forming process causes a primary benefit of rapid heating to a desired temperature for either warm or hot forming process, while averting the demand for expensive heating furnaces and long heating/cycle period [4].

The EAF technique has received increasing attention and is gradually used in the manufacturing process at lower cost of time and energy compared with traditional hot forming of titanium alloys. Ross et al. [5] investigated the use of direct current with various current densities to aid in the bulk deformation of TC4 titanium alloy under tensile and compressive loads. In both cases, electrical current significantly decreases the load required to deform a given test specimen under uniform conditions. Using carefully designed experiments, this study also characterizes and isolates the effect of resistive heating from the overall effect due to the electrical flow. It clearly indicates that the electricity’s effect is not primarily due to resistive heating. Jiang et al. [6] conducted both electrically assisted tension and pre-treatment tension to compare different effects on deformation resistance reduction and ductility improvement of TC4 titanium alloy. Corresponding isothermal tests including thermally assisted tension, as well as pre-treatment tension were also performed to distinguish the thermal effect from electroplastic effect both experimentally and numerically. The result indicates that the elevated temperature alone is insufficient to account for additional stress drop in the initial yield stress. The electric current is more effective in accelerating phase transformation from α phase to β phase than that obtained in oven-heat treatment at similar temperature. However, Magargee et al. [7] conducted uniaxial tension tests on thin commercially pure titanium sheets subjected to electrically assisted deformation using a new experimental setup to decouple thermal-mechanical and possible electroplastic behavior. When the tension specimens were air-cooled to near room temperature, the stress reductions were not observed and thus the role of temperature on the plastic behavior of metals subjected to electrically assisted deformation was reevaluated. Similarly, Kinsey et al. [8] found that an electroplastic effect did not exist during tension Kolsky bar experiments on 1 mm thick TC4 samples with up to 180 A/mm^2^ applied. The formability improvements observed during electromagnetic forming are not related to electroplastic effects from the induced eddy currents in the workpiece. This could be due to the theory of viscous drag of dislocation in the lattice structure, which has been hypothesized but is difficult to demonstrate experimentally. According to previous research results, it is still unrevealed how electric current influences the deformation behavior of TC4 titanium alloy. Nevertheless, some studies on EAF processes of TC4 titanium alloy were performed. Li et al. [9] conducted electrically-assisted 90°V bending tests of TC4 titanium alloy sheet. The bending crack can be avoided with the pulse current, which always appears without a current. Zhao et al. [10] investigated the effect of electric pulse on the springback during stretch U-bending of TC4 titanium alloy sheets. It is found that the introduction of electric pulses can effectively reduce the springback effect of TC4 and provide an alternative method to traditional hot forming process in realizing precise forming. 

The development of aircraft technique requires integral manufacturing of a large thin-wall component of TC4 titanium alloy. Its length reaches to above 10 m. It is completely impossible to produce the large component by the traditional hot forming process since ultra-large heating furnace and forming equipment are necessary. However, the EAF technique combined with an incremental forming process is very hopeful since the thin blank is directly heated by electric current and progressively formed. In this work, the aim is to validate the feasibility of integral manufacturing the large thin-wall component by electrically-assisted incremental forming process. Two-step finite element simulation by commercial software was chosen to predict the deformation behaviors. Subsequent electrically-assisted incremental forming tests were conducted to reveal the relation between process parameters and forming quality.

## 2. Numerical Simulation

The simulation includes two steps, i.e., thermal-electricity coupling simulation and thermo-mechanical coupling simulation. The two-step simulation is correlated by an analytical field transfer. The advantage of the step-by-step simulation method is to select different element types. The solid element for the sheet is chosen to simulate the current flow direction and temperature distribution during thermal-electricity coupling simulation. In order to improve the calculation efficiency of thermo-mechanical coupling simulation, the shell element is selected for the sheet. In the first step, the temperature field distribution is calculated under different pulse current densities. Since the solid element in the first simulation step is changed to the shell element in the second step, the calculation results in thermal-electricity coupling simulation cannot be directly set as the initial temperature conditions of thermo-mechanical coupling simulation. Therefore, the temperature data of sheet meshes are transformed to an analytic field, which is then mapped to geometric model of the sheet in the subsequent step. The deformation procedure and springback values during electrically-assisted forming are finally predicted.

**Figure 1:**
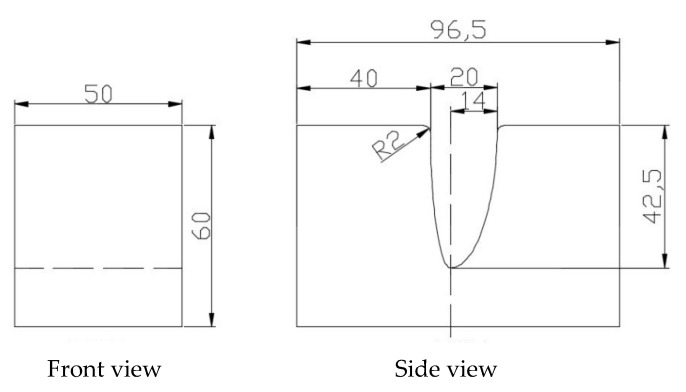
The geometry of forming die.

Figure 1 shows the forming die geometry. The clearance between the die and punch is 1.1 t (t refers to TC4 sheet thickness, 0.5 mm). The sheet width is 50 mm, which is four times wider than that of the punch. The mechanical parameters and thermal conductivity of TC4 titanium alloy vary with the increase of temperature. The data of yield stress at different temperatures (20~1100 °C) are used to depict the flow behaviors of TC4 alloy [2,9,11,12]. The thermal conduction coefficients at different temperatures are also inputted into the simulation model [13]. In the first step simulation, a linear thermal-electricity coupling hexahedron element is chosen for the sheet. The loading nodes of pulse current are located at the contact zone of the punch and sheet. The contact locations change during the electrically-assisted incremental forming process. Thus, the current loading nodes move along the sheet width direction. In Abaqus software, a setting current value, which equals the total current amount divided by node number, is charged for every node. Additionally, the thermal exchange process of the sheet to the environment and tools are very complicated. Furthermore, the contact area of the sheet to the tools constantly changes during the forming process. Consequently, a relatively simple method of temperature boundary, i.e., heat sink method, is defined. The highest temperature during the sheet charging with pulse current is approximately calculated by this method. Two ends of the sheet are set as 20 °C to roughly simulate their heat conduction process, at which the Young’s modulus of TC4 alloy is 67 GPa. Additionally, the friction model based on penalty function is selected. The friction coefficient is set as 0.1. Incidentally, the punch velocity is set as 1 mm/s.

**Figure 2:**
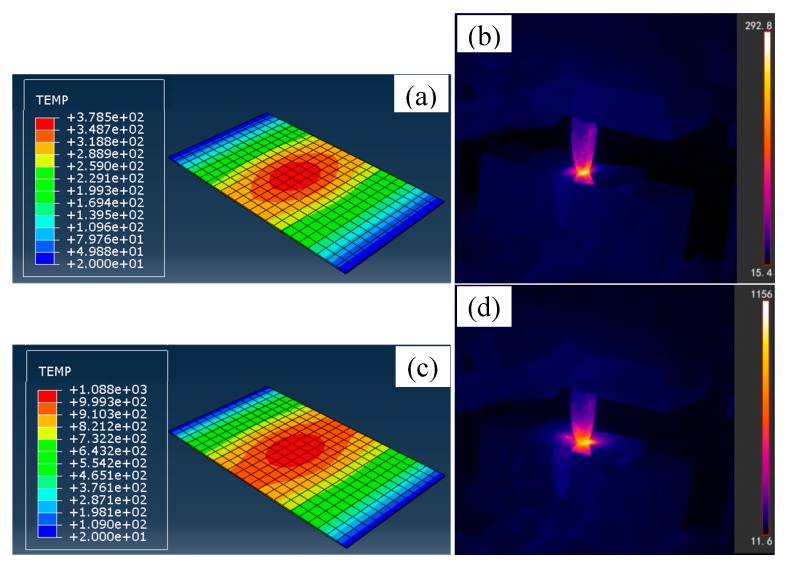
The predicted and measured sheet temperature distributions at different average current densities: (**a**) 4.8 A/mm^2^, prediction, (**b**) 4.8 A/mm^2^, measurement, (**c**) 10.9 A/mm^2^, prediction, (**d**) 10.9 A/mm^2^, measurement.

Figure 2 shows the sheet temperature distributions by prediction and measurement at different current densities. The temperature non-uniformly distributes. The pulse current density plays a key role in temperature increase. The contact area between the sheet and punch reaches the highest temperature, at which the largest current density exists. It obviously rises with increasing the pulse current density. When the average current densities are 4.8, 6.0, 7.9, and 10.9 A/mm^2^, the predicted highest temperatures reach 378.5, 518.9, 736.6, and 1088 °C, respectively. Actually, the temperature variations are measured by a FLIR infrared thermal camera. The measured temperature distribution trend is similar as the predicted result. Almost no current flows through the excluded contact areas between the sheet and die, in which the temperature increase mainly results from heat conduction and has indirect relation to the current amount. Due to poor thermal conductivity of TC4 titanium alloy, the temperature in these areas barely rises and approaches room temperature. 

**Figure 3:**
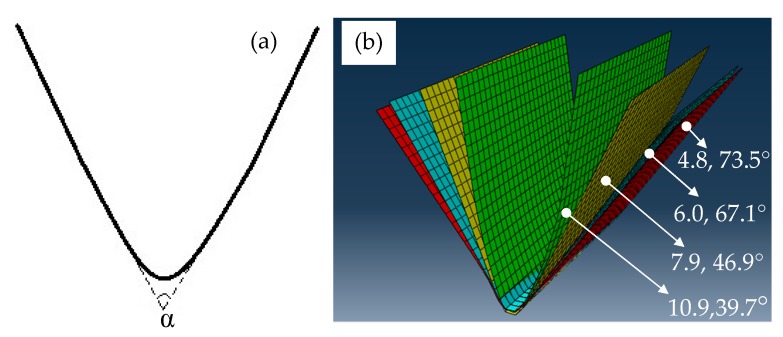
The springback angles of formed parts by prediction after thrice electrically-assisted forming at different average current densities: (**a**) Springback angle definition, (**b**) springback angles at different average current densities.

Figure 3 shows the springback angles of formed parts by numerical simulation after thrice electrically-assisted forming at different average current densities. For convenience, the included angle (α) of two sides of the formed part is defined as the springback angle. With increasing the pulse current density, the springback angle continuously declines. It mainly results from the joule heating effect of pulse current. The temperature increase can reduce the flow stress of TC4 titanium alloy. Then, the plasticity is obviously improved and the springback after electrically-assisted forming is also effectively restrained. After thrice forming, the predicted geometry nearly satisfies the designed demand. 

## 3. Experimental Validation

The electrically-assisted incremental forming tool was designed and manufactured. The width of the punch is 10 mm, which is a fifth of the sheet width. The sheet was placed on the die. The punch was moved down until it contacted the sheet. Then, the pulse power was turned on with a pulse current density range of 4.0~10.9 A/mm^2^ at a frequency of 140 Hz. A current circuit was consisted among the punch, sheet, and die. The central zone of the sheet was firstly formed. Subsequently, two zones of 15 mm distance to the sheet center were sequentially formed by moving the sheet. After thrice forming, the springback angles were evaluated.

**Figure 4:**
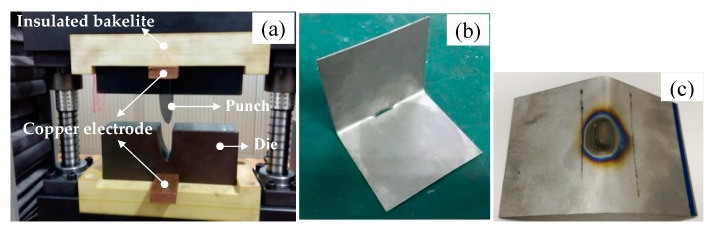
Electrically-assisted forming setup and firstly formed parts: (**a**) Forming setup, (**b**) no pulse current, (**c**) 10.9 A/mm^2^.

Figure 4 shows the firstly formed parts by electrically-assisted forming. The crack easily occurs without the pulse current. When the pulse current is charged at 10.9 A/mm^2^, the crack is completely avoided. However, the serious oxidation happens due to the temperature increase. The measured highest temperature reaches 1156 °C, which approaches the predicted value. It indicates that the joule heating effect maybe plays a dominant role in softening the TC4 titanium alloy. The formability of TC4 titanium alloy is remarkably improved. Actually, it is obtained in previous electrically- assisted uniaxial tension tests that the tensile strength decreases from 1100 MPa at room temperature to 743 MPa at 4.2 A/mm^2^. Meanwhile, the elongation increases from 12% to 12.5%. When the current density further increases to 9.6 A/mm^2^, the tensile strength decreases to 435 MPa and the elongation increases to 36% [9]. The flow stress reduction and plasticity improvement are helpful for restricting the springback.

Table 1 reveals the effects of forming times and current density on springback angles. In order to manufacture large TC4 parts, multiple forming is cleverly selected. The springback angles vary during sequential forming. In this study, the springback angles are obviously reduced with increasing the forming times and pulse current density. The formed part keeps close to the designed geometry. When the current density increases from 6.0 to 7.9 A/mm^2^ during thrice forming, the descent springack angle is 20°. However, the springack angle is only decreased by 3° with further increasing to 10.9 A/mm^2^. With increasing the forming times at 10.9 A/mm^2^, the descent springack angles decline from 18 to 8°. Therefore, three forming times can be almost completed for the set part at 10.9 A/mm^2^. This indicates that the electrically-assisted incremental forming process is feasible for integral manufacturing of large TC4 titanium alloy components.

**Figure 5:**
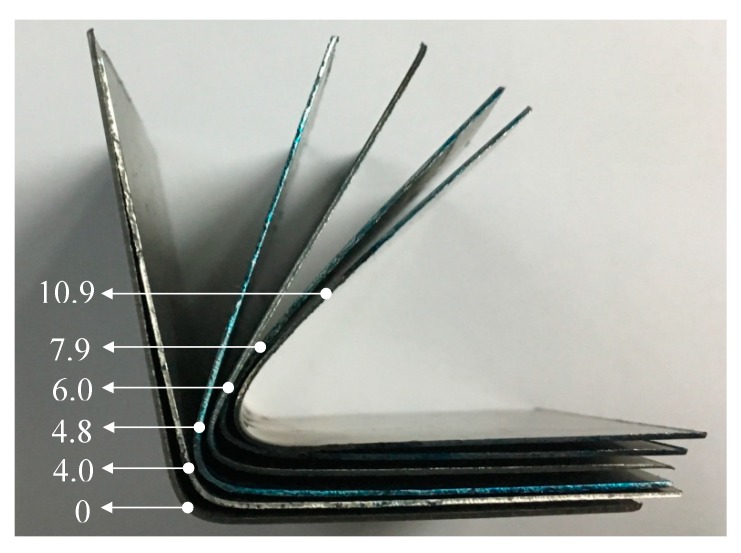
The formed parts after thrice forming at different current densities.

Figure 5 shows the formed parts after thrice forming at different current densities. The formed parts keep close to the ideal geometry with increasing pulse current densities, which accords to the above result. Nevertheless, it is noted that the shape of the formed part at 4.0 A/mm^2^ is nearly the same as that formed without the pulse current at room temperature. Additionally, the crack often happens at 4.0 A/mm^2^. The electroplastic effect hardly plays a role in improving the deformation ability of TC4 sheet. The springback angle prominently reduces when the current density increases to 4.8 A/mm^2^. Therefore, the current density threshold value maybe exists between 4 and 4.8 A/mm^2^ for the electroplastic effect of TC4 titanium alloy. Previous studies have highlighted a “threshold effect” whereby electrical application benefits are neglected until a critical current density is reached [14]. It was also found in copper alloy [15], 6061-T6 aluminum alloy [16], and AZ31B magnesium alloy [17]. The threshold effect maybe results from sufficient current being applied to aid dislocation motion past grain boundaries and element barriers [15]. 

**Figure 6:**
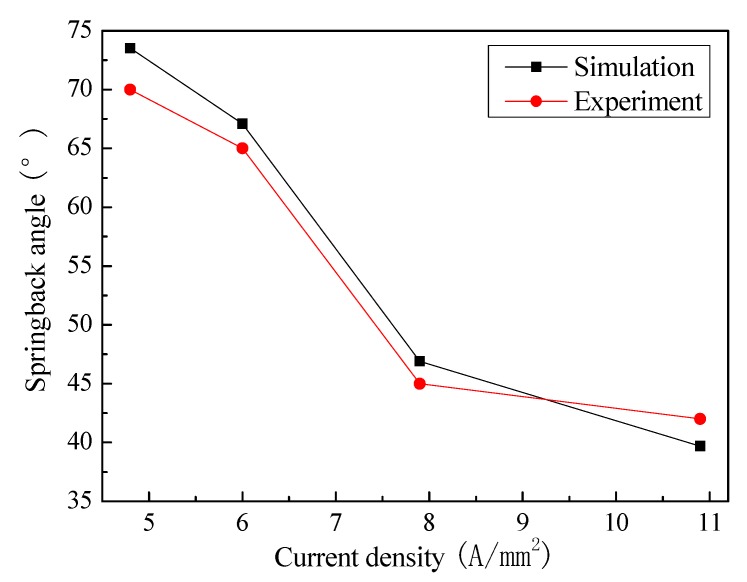
The springback angle comparisons between simulation and experiment.

Figure 6 shows the springback angles after thrice forming by simulation and experiment. Overall, the springback angles firstly reduce obviously when the current density increases from 4.8 to 7.9 A/mm^2^. Then, they slightly decline with further increasing the current density. Furthermore, the simulated value is close to the measured one. The highest error is only 6%. It indicates that the two-step simulation is feasible for predicting the springback characteristics of electrically-assisted incremental forming. Nevertheless, the error mainly results from element size, thermal boundary condition, and experimental measurement. The sheet element size during the forming simulation should be refined in the radius zone, which is helpful for improving the simulation accuracy. The heat sink method is used to simplify thermal exchange between the sheet and surrounding environment. In fact, the temperature calculation error exists as shown in Figure 2, which also affects the springback angle prediction accuracy. Additionally, the two sides of the formed part are curved, which results in the measurement error of springback angle.

## 4. Conclusions

The electrically-assisted incremental forming process of thin TC4 titanium alloy sheet is studied by numerical simulation and experiment. Some conclusions can be drawn as follows: 

(1) The two-step simulation including thermal-electricity coupling simulation and thermo-mechanical coupling simulation can effectively predict the springback characteristics of electrically- assisted incremental forming of thin TC4 titanium alloy sheet. The highest error is 6% for springback angle calculation.

(2) With increasing the forming times and pulse current density, the shape of the formed part keeps close to the ideal geometry. In this study, the thrice forming at 10.9 A/mm^2^ satisfies the precision requirement. It is feasible for integral manufacturing of large titanium alloy part by an electrically-assisted incremental forming technique.

## Figures and Tables

**Table 1:** The effects of forming times and current density on springback angles.

Forming Times	Springback Angles at Different Current Densities (°)			
	4.8 A/mm^2^	6.0 A/mm^2^	7.9 A/mm^2^	10.9 A/mm^2^
1	86	86	75	68
2	71	70	55	50
3	70	65	45	42
